# Evolution of late steps in exocytosis: conservation and specialization of the exocyst complex

**DOI:** 10.12688/wellcomeopenres.15142.2

**Published:** 2019-11-29

**Authors:** Cordula Boehm, Mark C. Field

**Affiliations:** 1 School of Life Sciences, University of Dundee, Dow Street, Dundee, DD1 5EH, UK; 2 Biology Centre, Institute of Parasitology, Czech Academy of Sciences, České Budějovic, 37005, Czech Republic

**Keywords:** Exocytosis, exocyst, eukaryotes, membrane transport, molecular evolution, comparative genomics

## Abstract

**Background:** The eukaryotic endomembrane system most likely arose *via* paralogous expansions of genes encoding proteins that specify organelle identity, coat complexes and govern fusion specificity. While the majority of these gene families were established by the time of the last eukaryotic common ancestor (LECA), subsequent evolutionary events has moulded these systems, likely reflecting adaptations retained for increased fitness. As well as sequence evolution, these adaptations include loss of otherwise canonical components, the emergence of lineage-specific proteins and paralog expansion. The exocyst complex is involved in late exocytosis and additional trafficking pathways and a member of the complexes associated with tethering containing helical rods (CATCHR) tethering complex family. CATCHR includes the conserved oligomeric Golgi (COG) complex, homotypic fusion and vacuole protein sorting (HOPS)/class C core vacuole/endosome tethering (CORVET) complexes and several others. The exocyst is integrated into a complex GTPase signalling network in animals, fungi and other lineages. Prompted by discovery of Exo99, a non-canonical subunit in the excavate protist *Trypanosoma brucei,* and availability of significantly increased genome sequence data, we re-examined evolution of the exocyst.

**Methods:** We examined the evolution of exocyst components by comparative genomics, phylogenetics and structure prediction.

**Results:** The exocyst composition is highly conserved, but with substantial losses of subunits in the Apicomplexa and expansions in Streptophyta plants, Metazoa and land plants, where for the latter, massive paralog expansion of Exo70 represents an extreme and unique example. Significantly, few taxa retain a partial complex, suggesting that, in general, all subunits are probably required for functionality. Further, the ninth exocyst subunit, Exo99, is specific to the Euglenozoa with a distinct architecture compared to the other subunits and which possibly represents a coat system.

**Conclusions:** These data reveal a remarkable degree of evolutionary flexibility within the exocyst complex, suggesting significant diversity in exocytosis mechanisms.

## Introduction

**Figure 1.**
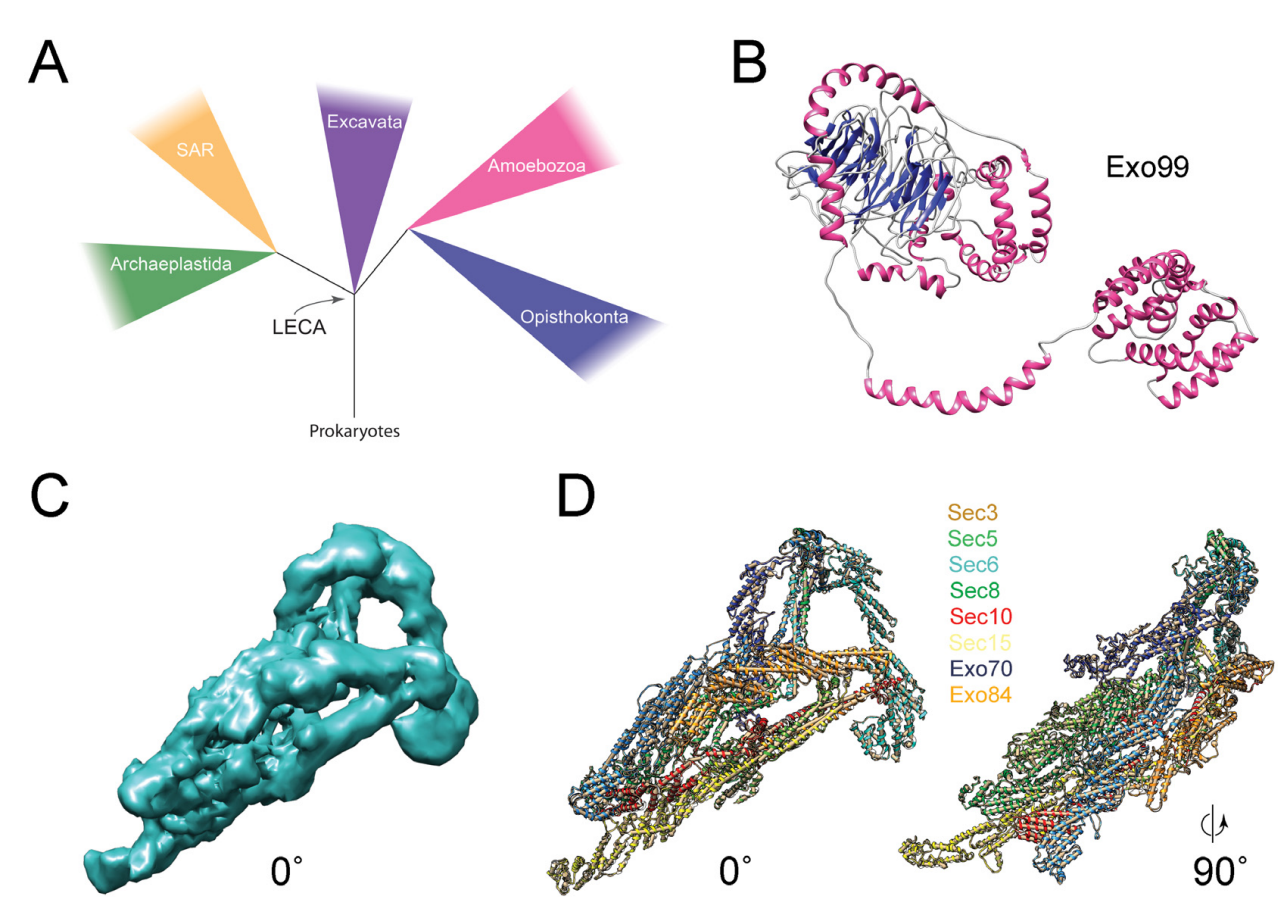
Evolution and structure of the exocyst. **A**) Cartoon representing the major supergroups, which are referred to in the text. The inferred position of the last eukaryotic common ancestor (LECA) is indicated and the supergroups are colour coordinated with all other figures. **B**) Structure of trypanosome Exo99, modelled using Phyre2 (intensive mode). The model for the WD40/b-propeller (blue) is likely highly accurate. The respective orientations of the a-helical regions may form a solenoid or similar, but due to a lack of confidence in the disordered linker regions this is highly speculative. **C** and **D**) Structure of the *Saccharomyces cerevisiae* exocyst holomeric octameric complex. In **C** the cryoEM map (at level 0.100) is shown and in **D**, the fit for all eight subunits (pdb 5yfp). Colours for subunits are shown as a key, and the orientation of the cryoEM and fit are the same for **C** and **D**. All structural images were modelled by the authors from PDB using UCSF Chimera.

A sophisticated level of cellular compartmentalisation is the major feature that differentiates prokaryotic from eukaryotic cells and underpins the origins of the nucleus. Early eukaryotic ancestors possessed a complex internal membrane system, suggesting rapid evolution after the first eukaryotic common ancestor (FECA) arose and prior to origin of the major eukaryotic super-groups ( Dacks & Field, 2018; Guy *et al.*, 2014; Koumandou *et al.*, 2013; Schlacht *et al.*, 2014). It is clear that these systems predate the origins of what would be classically recognised as eukaryotes, as some ancestral genes for constructing an endomembrane system were present in prokaryotes, and specifically Archaea ( Eme *et al.*, 2018; Spang *et al.*, 2018) ( Figure 1A).

An established theme in the evolution of membrane transport is the central role of paralogous protein families in dictating compartmental identify, specificity and supporting transport functions between compartments. These families include small GTPases, SNAREs, coat complexes and tethers. For example, SNARE and Rab paralogs associate with distinct subcellular organelles ( Elias *et al.*, 2012; Khurana *et al.*, 2018; Zerial & McBridge, 2001), and it is likely that new organelles and/or pathways develop via emergence of novel SNARE and/or Rab paralogs through gene duplication and neofunctionalisation ( Dacks & Field, 2007; Ramadas & Thattai, 2013). The evolution of some of these families has been reconstructed in some detail ( Elias *et al.*, 2012; Hirst *et al.*, 2014; Venkatesh *et al.*, 2017).

Deep evolutionary relationships between proteins forming vesicular coats and other structures, including the COPI and II complexes, clathrin/adaptin heterotetramers and the nuclear pore complex, further supports the concept of stepwise acquisition of complexity prior to the last eukaryotic common ancestor (LECA) ( Rout & Field, 2017). Perhaps most remarkable is the presence of a fully differentiated set of coat complexes and specificity-encoding machinery in the LECA, and consequentially, over a billion years this ancestral endomembrane system has expanded, contracted and neofunctionalised such that the current configurations of eukaryotic endomembrane systems vary hugely. More recently it has been speculated, based on the diversity of the architecture of nuclear pore complex subunits, that the nucleus, and possibly the intraflagellar transport system, arose comparatively late, during the transition from FECA to LECA ( Field & Rout, 2019).

A further group of proteins central to compartmentalisation are the membrane-tethering complexes (MTCs). Considerably more diverse than Rab and SNARE families in both architecture and mechanism(s) of action, MTCs control Rab GTP cycles, as well as tether vesicles for fusion. MTCs have splendid names that include transport protein particle (TRAPP) I, II and III, conserved oligomeric Golgi (COG), homotypic fusion and vacuole protein sorting (HOPS), class C core vacuole/endosome tethering (CORVET) (plus class C homologs in endosome-vesicle interaction, CHEVI and factors for endosome recycling and retromer interactions, FERARI), dorsalin-1 (Dsl1), Golgi-associated retrograde protein/endosome-associated recycling protein (GARP/EARP) and the exocyst. Significantly, MTCs vary considerably in the number of subunits they possess, but evidence for common evolutionary descent for some MTC subunits has been offered ( Koumandou *et al.*, 2007; Whyte & Munro, 2002; Yu & Hughson, 2010). MTCs are widely distributed among eukaryotic taxa and many subunits share the complexes associated with tethering-containing helical rods (CATCHR) fold, consistent with a common origin for MTCs ( Klinger *et al.*, 2013; Koumandou *et al.*, 2007; Yu & Hughson, 2010), and further supported by the structural similarity of several exocyst subunits sharing the CATCHR fold, which is almost exclusively α-helical ( Sivaram *et al.*, 2006; Vasan *et al.*, 2010). Further, the *Saccharomyces cerevisiae* CATCHR complexes, GARP, COG1–4 subcomplex of COG and HOPS share similar subunit organization ( Chou *et al.*, 2016). This is not only consistent with possible common ancestry, but also may indicate mechanistic similarities.

Exocyst subunits were initially identified as Sec3, 5, 6, 8, 10 and 15 mutants in a screen for trafficking defects ( Novick *et al.*, 1980). Two additional subunits, Exo70 and Exo84, were subsequently discovered and the holocomplex presented as a stable 19.5S particle ( Bowser *et al.*, 1992; Guo *et al.*, 1999; TerBush *et al.*, 1996) ( Figure 1D). Overall, the yeast exocyst forms a loose open rod, but has considerable conformational flexibility ( Heider *et al.*, 2016; Picco *et al.*, 2017; TerBush *et al.*, 1996) and interacts with multiple plasma membrane-located GTPases ( Wu *et al.*, 2008) and can act as an effector to both Rho and Rab GTPases. CryoEM structures of the complex and subunits at 4.4Å resolution revealed a highly conserved architecture for the subunits with between two and four helical ‘CorEx’ bundles, together with an extended N-terminal α-helix that is critical for assembly ( Mei *et al.*, 2018). Notably, CorEx shares structural similarities with the N-terminus of COG and GARP subunits. Only Sec3 and Exo84 have an additional domain, namely a pleckstrin homology (PH) lipid interaction domain.

Both structural and experimental data from yeast suggests that the exocyst is formed of two heterotertrameric subcomplexes; Sec3, 5, 6, 8 and Sec10, 15, Exo 70 and 84. Sec3 appears critical for both assembly and disassembly ( Ahmed *et al.*, 2018; Luo *et al.*, 2014) and Exo84 phosphorylation is implicated in controlling overall exocyst assembly and function. Significantly, these Sec3 and Exo84 are components of different subcomplexes and likely interact with phospholipids through their PH domains. The exocyst has clear roles in secretion but is also implicated in disease susceptibility, host cell invasion by intracellular bacteria and development ( Arasaki *et al.*, 2018; Bonnemaijer *et al.*, 2018; Lira *et al.*, 2018) with evidence for additional roles in endocytosis/recycling also published ( Boehm *et al.*, 2017; Jose *et al.*, 2015; Monteiro *et al.*, 2013). Also the tetrameric subcomplexes function in autophagy with potential for additional specialisation ( Bodemann *et al.*, 2011; Kulich *et al.*, 2013).

Previous comparative genomics studies identified only six of the eight canonical exocyst subunits, with Sec5 and Exo84 evading identification in all trypanosomatids ( Koumandou *et al.*, 2007), suggesting a simplified exocyst complex in trypanosomatids, possible replacement of otherwise canonical subunits or failure to uncover highly divergent orthologs. The latter possibility was demonstrated following biochemical identification of all eight canonical subunits as well as a ninth, Exo99, in trypanosomes ( Boehm *et al.*, 2017). Using updated methodology and genome resources, we find evidence for considerable evolutionary flexibility in exocyst subunit retention, with essentially complete loss from some lineages and a tentative suggestion of a connection to novel coat proteins.

## Results

### Identifying exocyst subunits across the eukaryotes

The earlier failure to identify Sec5 and Exo84 in excavates by comparative genomics ( Koumandou *et al.*, 2007), and subsequent identification in trypanosomes by immunoisolation and mass spectrometry, indicated that this earlier study lacked sensitivity, and suggested other false negatives within the dataset ( Boehm *et al.*, 2017). Furthermore, the distribution of the recently identified Exo99 subunit has not been investigated systematically. Considerable genome sequencing has taken place in the period since the earlier analysis, as well as availability of superior search algorithms, prompting this reanalysis.

**Figure 2.**
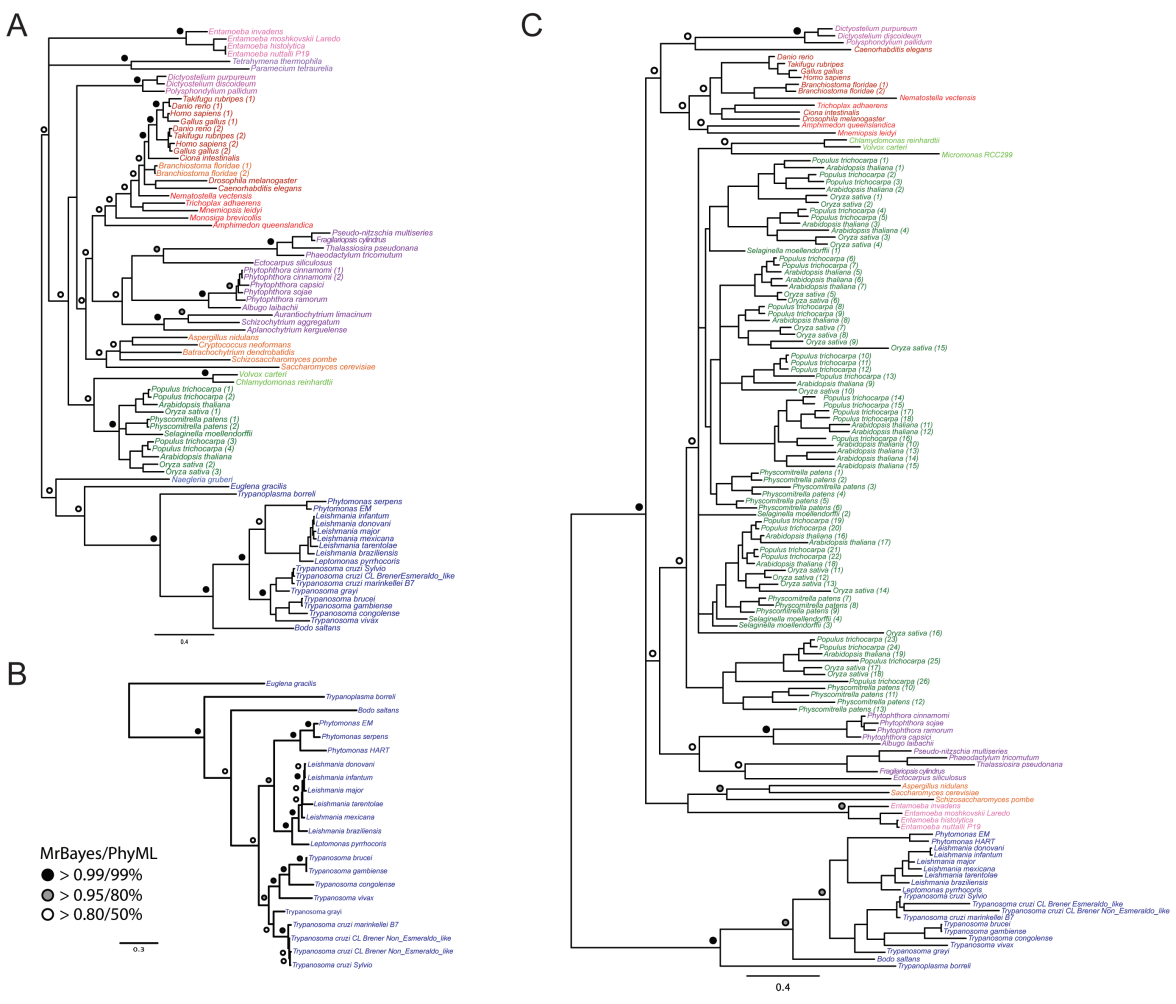
Phylogenetic reconstructions for Sec15, Exo99 and Exo70. Trees show the best Bayesian topology for reconstructions of **A**) Sec 15, **B**) Exo99 and **C**) Exo70. Numerical values at the nodes indicate statistical support from analysis with MrBayes and PhyML. Values for highly supported nodes have been replaced by symbols as indicated in the legend. Species names are coloured for recognised supergroups: Opisthokonta (blue), Amoebozoa (pink), Archeoplastida (green), Stramenopile-Alveolate-Rhizaria (SAR, orange), Excavata (purple). The same colour convention is used throughout this figure and phylogenetic trees.

**Figure 3.**
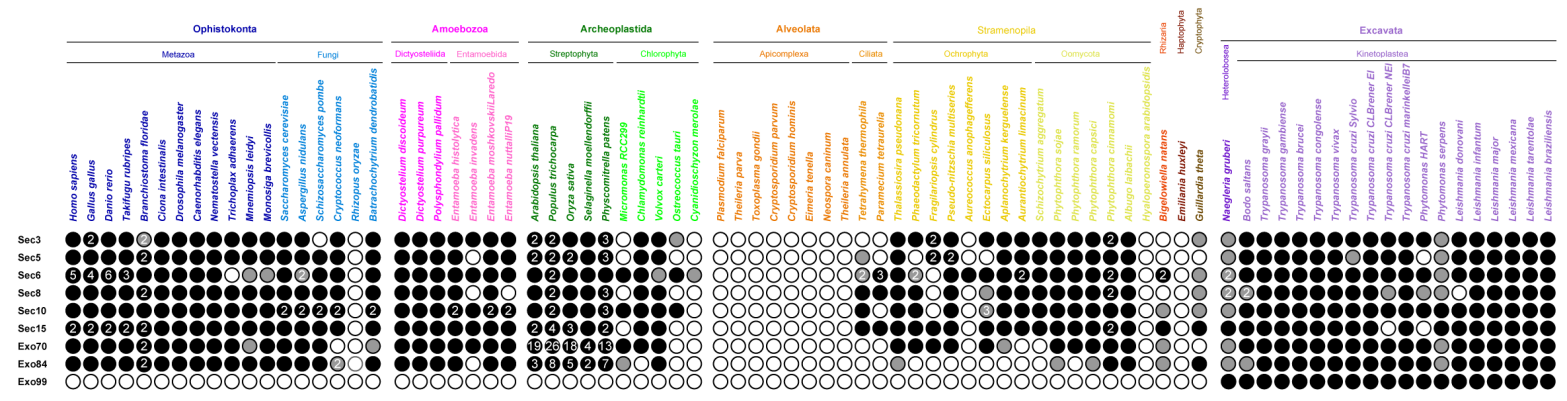
Coulson plot of distribution of exocyst components across eukaryotic lineages. Calls are based on a combination of BLAST, alignments and phylogenetic reconstruction. Filled circles indicate the presence of the protein, open circles that an ortholog was not found and grey indicates that the sequence could not be fully verified by phylogenetics. The numbers in the circles indicate multiple parlogs and the number of individual genes identified. Accession numbers for all reported exocyst subunits are given in Table S1 ( *Extended data*). Species names are coloured by supergroup as in Figure 2. The eight canonical exocyst subunits are conserved throughout the eukaryotic lineage, with the exception of the Alveolata. Some of the plants have greatly increased numbers of Exo84 and especially Exo70 genes in their genomes. The presence of Exo99 is restricted to the Excavata.

We screened for genes encoding the eight canonical exocyst subunits and the newly identified subunit Exo99 in 87 eukaryotic genomes by BLAST, inspection of alignments and phylogenetic reconstruction. This increased the size of our genome panel approximately five-fold and took advantage of the increased quality of these resources. Furthermore, we were able to harness high quality phylogenetic reconstruction to validate our data. Only subunit predictions that passed reciprocal BLAST, phylogenetic validation and were predicted to be within a similar length as the query, together with homology that extended over more that 50% of the sequence (to avoid calls based exclusively on conservation of small architectural features) were annotated as ‘found’. Example phylogenetic trees for three subunits (Sec15, Exo99 and Exo70) are shown in Figure 2 and the overall distribution in Figure 3. Phylogenetic trees for the remaining subunits, as well as accession numbers of identified orthologs are included as *Extended data* (Figures S1-6, Table S1).

### Distribution of the canonical octamer subunits

The eight canonical exocyst subunits are well conserved, reflecting their ancient origins and importantly are detected in representatives of all five eukaryotic supergroups. Phylogenetic analysis indicates that these sequences are *bona fide* orthologs and, as the topology of the gene and taxon trees are highly similar, there is no evidence for lateral gene transfer ( Figure 2). Given the noted similarities in structures of these subunits and the clear sharing of extensive α-helical bundles revealed by cryoEM, this suggests that the exocyst octamer indeed arose prior to the LECA and most likely by paralog expansion from an ancestral subunit complement.

We found few examples of partial retention of subunits and which may also indicate that some of our examples of single subunit losses are artifactual. This pattern of retention may also argue against fully independent functionality for the two subcomplexes and overall is consistent with recent functional studies ( Ahmed *et al.*, 2018).

We found expansions of Sec6 and 15 in vertebrates and the close relatives *Brachiostoma* (commonly lancelets), where multiple subunits have expanded. This pattern may reflect tissue complexity, but the absence of an obvious interaction between Sec6 and 15 suggests this is unlikely to be associated with a specific subfunction. However, the locations of these two subunits, located at opposite ends of the octameric complex, may suggest that this facilitates variation in interactions between exocytic vesicles and plasma membrane docking sites in different tissues ( Heider *et al.*, 2016; Heider & Munson, 2012). Sec10 is also expanded in the fungi, and there is complete absence of the complex in Rhizopus, the only such example in the Opistokhonta sampled here. As this fungal taxon lacks an ability for septation, this may explain the loss of the entire exocyst, although this is highly speculative.

Both major Amoebozoa lineages retain a full complement of exocyst subunits in the genomes of the majority of species sampled. The absence of several subunits from *Entamoaeba invadens* and one from *E. mutabilli* is noteworthy, as is the expansion of Sec10, a feature shared with fungi. While this could raise the possibility that a Sec10 duplication occurred at the root of the unikhonts, this is not supported by phylogenetic reconstruction, which suggests independent, *albeit* likely basal, origins for fungal and Entamoeba Sec10 paralogs (Figure S5, *Extended data*).

The most significant subunit expansion within the entire dataset is within the Streptophyta plants. Interestingly, in the closely allied algal Chlorophytes, loss is the dominant evolutionary trend, with *Cyanidioschyzon merolae* and *Ostreococcus tauri* lacking sufficient subunits to build a canonical exocyst, which may suggest alternate functions or mechanisms, at least for *O. tauri* and *Micromonas* where only four subunits could be identified. Interestingly, for Chlorophytes lacking many subunits, Sec6 and Sec10 are retained, which significantly are components of distinct subcomplexes and unlikely to physically interact ( Ahmed *et al.*, 2018). A small number of plants also have multiple Sec10 paralogs and, in common with other taxa (see above), the origin of the duplication was likely taxon-specific (Figure S5, *Extended data*).

The most extreme expansions within Streptophyta subunits are Exo70 and Exo84, with at least 26 copies detected in *Populus trichocarpa* and 18 in rice. We are aware that the total number of genes we have predicted for all eight exocyst subunits in plants varies to some extent from those previously published, mostly because we did not distinguish between genes with ≥99% sequence homology. However, this does not affect the overall consensus between our and previous studies; namely, that all exocyst subunits in plants are expanded to some extent.

Phylogeny indicates a complex evolutionary pathway for Exo84, and while our reconstruction suggests that most paralogs arose *via* lineage-specific expansions, the absence of good statistical support makes this conclusion equivocal (Figure S6, *Extended data*). By contrast, it is clear that Sec15 and Exo70 expansions began at the root of the Streptophyta and, in the case of Exo70, this has continued in a lineage-specific manner to create a family of paralogs of considerable diversity ( Figure 2). Land plant Exo70 paralogs can be grouped into three clades ( Cvrčková *et al.*, 2012; Synek *et al.*, 2006), which indicates an early establishment of these subfamilies. Live imaging in mammalian cells suggests that Exo70 is the first subunit to contact the plasma membrane ( Ahmed *et al.*, 2018), and hence the presence of so many Exo70 variants is likely a result of tissue-specific and/or plasma membrane-domain targeting specificity. In plant cells the presence of multiple cortical subdomains and differential interactions with Exo70 paralogs has been described, as well as the presence of multiple Exo70 paralogs within a single cell ( Synek *et al.*, 2014; Žárský *et al.*, 2009; Žárský *et al.*, 2013). Thus multiple factors likely underpin the Exco70 expansions, including differential targeting, noncanomical functions as well as defence against pathogens ( Žárský *et al.*, 2019; Zhao *et al.*, 2015). The evolution of multiple trafficking pathways based around expansion of canonical systems also extends to other MTC-mediated pathways, specifically HOPS and CORVET ( Takemoto *et al.*, 2018).

All sampled Aplicomplexa, including *Plasmodium falciparum* and *Toxoplasma gondii*, ** lack the entire exocyst complex, while other alveolates within the Ciliata lineage have retained a subset of subunits. Since the retained subunits vary between the two ciliates analysed, including Exo70 and Exo84, this suggests individual losses rather than a stepwise loss of exocyst function during the evolution of the alveolates and raises the question for the existence of an exocyst-independent exocytic pathway in these organisms. It is, however, clear that loss from the Apicomplexa is an ancestral event. Apicomplexa are known for a patchy distributions of other tethering complexes like COG, GARP, Dsl1 and TRAPPII ( Koumandou *et al.*, 2007), and which may reflect simplifications of trafficking systems in these obligate intracellular parasites. Many Apicomplexa possess unique secretory organelles, including micronemes and rhoptries, that are essential for host cell invasion, but these organelles appear not to require the canonical MTC systems ( Tomavo, 2014).

Amongst Stramenopiles, there is also a complex pattern of retention and loss. There is near full retention amongst the Oomycota, which contrasts with the many losses in the sister taxon Ochrophyta. It may be significant that in these organisms, Exo84 and, in a more limited manner, Exo70 are most commonly absent, similar to the ciliates. A limited number of expansions are also detected, principally in Sec3, Sec5 and Sec6, which may suggest more diversity within vesicular cargo transport than at the plasma membrane, as all three of these subunits are components of a single subcomplex that likely interfaces with the incoming vesicle ( Ahmed *et al.*, 2018).

Only single orthologues of all exocyst subunits were found in the kinetoplastids, with possible duplications in *Bodo saltans* and *Trypanoplasma borelli*. The few apparent losses, for example in *T. cruzi* and *Phytomonas* HART are most likely the result of incomplete sequence data/assembly, with the suggestion that, for these taxa, the composition of the canonical octameric exocyst component is essentially invariant.

### Exo99, a taxon-restricted subunit with distinct structure

Exocyst subunits were originally identified *via* yeast secretory mutant screens, which uncovered six subunits (Sec3, 5, 6, 8, 10 and 15), and interaction between Sec15 and Sec4, a small GTPase at the plasma membrane required for secretion and orthologous to Rab11 ( Novick *et al.*, 1980) and with continually expanding roles in endosomal dynamics ( Zulkefli *et al.*, 2019). Two additional subunits, Exo70 and Exo84, were subsequently described, and the entire system demonstrated by biochemical and multiple interactome analyses to be a stable 19.5S complex, *albeit* with evidence for the presence of additional forms ( Bowser *et al.*, 1992; Guo *et al.*, 1999; Morgera *et al.*, 2012). A ninth subunit, Exo99, was identified by affinity isolation in African trypanosomes. Exo99 phenocopies Sec15 under knockdown, indicating that it is a *bona fide* member of the complex ( Boehm *et al.*, 2017). Exo99 is present in all kinetoplastids and related bodonids ( *Bodo saltans*), suggesting a unique aspect in export pathways in these organisms.

The structure of Exo99 is highly distinct from the canonical exocyst subunits and possesses a confidently predicted seven blade β-propeller at the N-terminus, together with an α-helical C-terminus ( Figure 1B). The topology of several short stretches of the C-terminal region is predicted as disordered, preventing assessment of the overall architecture of the α-helical region. Hence, it is unclear if this region adapts a fold similar to the CATCHR family and hence other exocysts subunits or is distinct. Very weak homologs were also found in *Naegleria gruberi, Trichomonas vaginalis, Giardia lamblia* and social amoeba. Structure prediction suggests that the *N. gruberi* sequence may well share architecture with the kinetoplastida (Data archive 2, Extended data), but that the other possible orthologs do not, indicating likely restriction to Euglenozoa, as well as possibly the heterolobosids.

It is tempting to speculate that Exo99 is a divergent member of the protocoatomer family, which populate the endomembrane system. These proteins are associated with vesicular transport and related functions and bear the β-propeller N-terminus as well as an α-helical C-terminal domain, perhaps best recognized in the heavy chain of the endocytosis coat protein clathrin ( Rout & Field, 2017). However, in that instance the helices form a coiled-coil solenoid, a specific type of higher order architecture, whilst for Exo99 it is unclear if this is the case. Clearly, more precise structural data are required to evaluate this possibility, as well as the location of Exo99 within the trypanosome exocyst. It is also unclear if additional coat-like components are associated with the trypanosomatid exocyst, but not captured in the affinity isolation. Most significant is that the presence of this divergent subunit, which evaded detection by *in silico* methods due to its novelty, opens the possibility of additional lineage-specific exocyst components in other species.

## Discussion

Cellular complexity was revolutionised by the development of an endomembrane system during eukaryogenesis. Subsequently losses and gains have moulded the ancestral transport system into the huge variety observed across the range of eukaryotes. Secondary loss of components is common, while expansion of individual paralogs is also a high frequency event. It is less common to uncover the birth of completely new components or complexes. Overall, this may well reflect the range of cellular processes with which the exocyst has been implicated, and while most of these are membrane transport or likely to be connected intimately, exocyst function may extend beyond to include, for example, translation ( Lipschutz *et al.*, 2003). The interaction of the exocyst with members of multiple subfamilies of the Ras GTPases, including Rabs, Arfs, Rho and others, together with compositional changes reflects this promiscuity, and which is, to some extent, also mirrored by the present study. The exocyst is an open, monomeric rod, with each component present as single copy, but several complexes appear to be required for vesicle fusion ( Ahmed *et al.*, 2018). All canonical subunits share the CorEx predominantly α-helical secondary structure suggesting a stepwise pathway for exocyst origins, and supported further by the share the CATCHR architecture of additional MTC subunits ( Sivaram *et al.*, 2006; Vasan *et al.*, 2010). 

**Figure 4.**
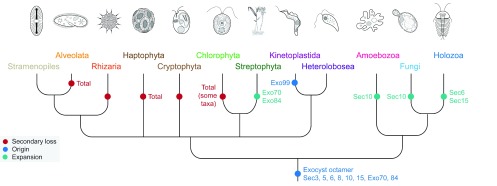
Schematic eukaryotic evolutionary tree illustrating the major shifts in exocyst complexity. Examples of individual lineages are shown at the top, and taxon names are colour-coded as before. Red dots indicate substantial losses, blue dots the origins of subunits and teal dots indicate major expansions. Many other smaller scale events, for example single subunit loss or duplication in a single species, are not illustrated for greater clarity.

There is considerable diversity in the retention of genes encoding exocyst subunits, with many examples of complete loss or spectacular expansion ( Figure 4). Considering subunit losses there are examples in fungi, plants and multiple protozoan lineages. Some, such as *C. merolae,* may reflect known unusual biology, and a complete absence of excess subunits in Apicomlexa is also consistent with the highly unusual and reduced secretory systems in a predominantly parasitic lineage. For example, there is repeated loss of adaptins and degeneration of the Golgi complex in Apicomplexa ( Nevin & Dacks, 2009), which correlates well with the absence of much of the COG, Dsl and TRAPPII complexes ( Klinger *et al.*, 2013), while loss of the exocyst dates back to the origins of the Chromista ( Woo *et al.*, 2015).

Expansions generally involve a subset of subunits, with Sec6, 10 and 15 duplicated across more than one supergroup, a pattern indicating independent events. Larger scale expansions are Exo70 and 84 in the higher plants. Significantly, Exo70 in *Arabidopsis thaliana* carries multiple motifs for interactions with Atg8 ( Cvrčková & Zárský, 2013) and Exo70 has been subjected to extreme paralogous expansion in Streptophyte plants and is likely the product of selective pressures ( Zárský *et al.*, 2009, Zárský *et al.*, 2013). Significantly, plants also possess expanded Rab11 (RabD) paralogs ( Rutherford & Moore, 2002), but a specific relationship between Rab11 and Exo70 paralogs has not been demonstrated. Retention of the exocyst is, in the main, all-or-nothing, indicating that the complex functions essentially as a single unit, and loss of even one subunit compromises function, consistent with the structure of the complex ( Ahmed *et al.*, 2018; Heider *et al.*, 2016; Mei *et al.*, 2018). Exo99 is currently the sole example of a lineage-specific exocyst subunit, and may be part of a larger coat complex, based on similarity to protocoatomer. The presence of Exo99 further highlights evolutionary modifications to membrane trafficking pathways and underscores the flexibility of these pathways across evolution, as well as suggesting that there may be additional exocyst components in other lineages ( Manna *et al.*, 2017; Rout & Field, 2017). Overall, despite considerable conservation, there is remarkable sculpting of exocyst complex composition, which suggests that, despite the already significant catalog of activities, a range of functional roles may remain to be uncovered. This significant evolutionary flexibility offers an interesting paradigm for unravelling the evolutionary pressures that have moulded eukaryote evolution.

## Methods

### Comparative genomics of exocyst components

Candidate exocyst components were identified by scanning a panel of eukaryotic predicted proteomes (Table S1 Extended data) with known exocyst component sequence queries using BLAST ( Altschul *et al.*, 1990). For each subunit, one query sequence was selected from each of the following predicted proteomes: *Homo sapiens, Saccharomyces cerevisiae, Trypanosoma brucei, Dictyostelium discoideum,* a chromalveolate ( *Phytophthora capsici/Albugo laibachii/Phytophthora sojae/Phytophthora ramorum*), an Archaeplastida *(Arabidopsis thaliana/Chlamydomonas reinhardtii/Selaginella moellendorffii*) (accession numbers for initial queries provided in Table S1). For each subunit, the top BLAST hits from each of these scans were pooled in a neighbour-joining tree after alignment with ClustalW with default parameters to remove erroneous sequences ( Thompson *et al.*, 1994). The gene IDs on the tree were then annotated with predicted protein length (based on) alignments with known exocyst components, pfam domain predictions (pfam server default parameters at https://pfam.xfam.org) and notes of which (if any) of the six initial query sequences detected the ID as a reciprocal best BLAST hit. This annotation allowed the identification of a cluster of robust candidates by neighbour joining (NJ). Off-target matches were identified by manual inspection of both the annotated NJ tree and the underlying alignment and these were excluded. Furthermore, the overall length of the predicted protein and the region of homology were considered, to exclude proteins that were likely only related through possession of a common domain.

In cases where a candidate was not found, additional datasets were queried by web-based BLAST searches at TriTrypDB, JGI and NCBI as appropriate. Alignments were created using MUSCLE ( Edgar, 2004). Only unambiguous homologous regions were retained for phylogenetic analysis, performed by two separate methods. To obtain the Bayesian tree topology and posterior probability values, the program MrBayes version 3.2.2 was used ( Ronquist & Huelsenbeck, 2003) running 8,000,000 generations. Maximum-likelihood (ML) analysis was performed using PhyML v3.0 ( Guindon & Gascuel, 2003) with 100 bootstrap replicates. Nodes with better than 0.95 posterior probability and 80% bootstrap support were considered robust, and nodes with better than 0.80 posterior probability and 50% bootstrap support are shown.

### Structure prediction

The structures of both *T. brucei and N. gruberi* Exo99 proteins were predicted using the Phyre2 server running under intensive mode ( Kelley *et al.*, 2015, full output available in Extended data). The data for this, as well as for the exocyst octameric complex of *S. cerevisiae,* were visualized using UCSF Chimera ( Pettersen *et al.*, 2004). Data for experimentally determined structures were retrieved from PDB ( https://www.rcsb.org).

### Graphics production

The Coulson plot in Figure 3 was produced using Coulson Plot Generator ( Field *et al.*, 2013) and binaries available at http://sourceforge.net/projects/coulson. All images were prepared for final production in Adobe Illustrator 23.0.3.

## Data availability

### Underlying data

All data underlying the results are available as part of the article and no additional source data are required. ** Extended data, including additional trees, accessions for all hits and structure prediction outputs from Phyre2 are available at https://figshare.com/articles/Extended_data/8167724
