# Supplementary material for: Evolution of late steps in exocytosis: conservation and specialization of the exocyst complex
Source: Wellcome Open Res. 2019 Nov 29;4:112. Originally published 2019 Jul 26. [Version 2] doi: 10.12688/wellcomeopenres.15142.2 (PMC6784791; doi:10.12688/wellcomeopenres.15142.2)
Supplement: Peer review history

# Peer review: Reviewer response for version 2

**DOI:** 10.21956/wellcomeopenres.17085.r37210

**Published:** 2019-12-02

**Authors:** Joshua H. Lipschutz

**Affiliations:** 1 Department of Medicine, Medical University of South Carolina (MUSC), Charleston, SC, USA

I have no further concerns.

I confirm that I have read this submission and believe that I have an appropriate level of expertise to confirm that it is of an acceptable scientific standard.

# Peer review: Reviewer response for version 1

**DOI:** 10.21956/wellcomeopenres.16521.r36430

**Published:** 2019-10-28

**Authors:** Joshua H. Lipschutz

**Affiliations:** 1 Department of Medicine, Medical University of South Carolina (MUSC), Charleston, SC, USA

The manuscript, “ *Evolution of late steps in exocytosis: conservation, specialization*”, addresses an interesting question, namely the evolution of the exocyst complex. I think the authors do a good job of describing the comparative genomics, phylogenetics and structure prediction of the exocyst.

 Where the authors could do better is describing the many cellular functions of the exocyst and offering a possible mechanism for this. For example, in the abstract the authors write, “The exocyst complex is involved in late exocytosis, and possibly additional pathways, and is a member…”. In the introduction, the authors describe some of the other additional pathways, “The exocyst has clear roles in secretion but is also implicated in disease susceptibility, host cell invasion by intracellular bacteria and development (Arasaki et al., 2018 ^1^; Bonnemaijer et al., 2018 ^2^; Lira et al., 2019 ^3^) with evidence for additional roles in endocytosis/recycling also published (Boehm et al., 2017 ^4^; Jose et al., 2015 ^5^)”. This is not a complete list as the exocyst has also been shown to be centrally involved in basolateral protein transport (Grindstaff et al, Cell, 1998 ^6^), ciliogenesis (Zuo et al, Mol Biol Cell, 2009 ^7^), and protein translocation in the ER (Toikkanen et al, J Biol Chem,  2003 ^8^;  Lipschutz et al, J Biol Chem,  2003 ^9^). How does the exocyst perform these myriad functions? The authors allude to a possible mechanism when they write, “Based on prolific interactions with multiple Ras-superfamily GTPases, the exocyst has been proposed to act as a tether to bring secretory vesicles to the plasma membrane, and recent in vivo imaging supports this model (Ahmed et al., 2018 ^10^; Picco et al., 2017 ^11^)”. The exocyst has actually been shown to be regulated by multiple GTPases of the Arf (e.g. Seixas et al, Mol Biol Cell, 2016 ^12^; ), Ral (e.g. Moskalenko et  al, Nat Cell Biol, 2002 ^13^; Sugihara et al, Nat Cell Biol, 2002 ^14^; Brymora et al, J Biol Chem 2001 ^15^), Rab (Knodler et al, PNAS, 2010 ^16^), and Rho (Phiel et al, J Biol Chem, 2001 ^17^; Choi et al, J Am Soc Nephrol, 2013 ^18^) families. A possible model is that the exocyst is like a Swiss army knife and has the ability to accomplish multiple tasks depending on which small GTPase is regulating it. I think this kind of discussion would strengthen the manuscript.

I confirm that I have read this submission and believe that I have an appropriate level of expertise to confirm that it is of an acceptable scientific standard, however I have significant reservations, as outlined above.

## References

[1] : Legionella remodels the plasma membrane–derived vacuole by utilizing exocyst components as tethers. *The Journal of Cell Biology*.2018;217(11) : 10.1083/jcb.201801208 3863-3872 10.1083/jcb.201801208 PMC621971730275106

[2] : Genome-wide association study of primary open-angle glaucoma in continental and admixed African populations. *Hum Genet*.2018;137(10) : 10.1007/s00439-018-1943-7 847-862 10.1007/s00439-018-1943-7 30317457PMC6754628

[3] : The Exocyst Component Exo70 Modulates Dendrite Arbor Formation, Synapse Density, and Spine Maturation in Primary Hippocampal Neurons. *Mol Neurobiol*.2019;56(7) : 10.1007/s12035-018-1378-0 4620-4638 10.1007/s12035-018-1378-0 30374940

[4] : SMYD2-Mediated Histone Methylation Contributes to HIV-1 Latency. *Cell Host Microbe*.2017;21(5) : 10.1016/j.chom.2017.04.011 569-579.e6 10.1016/j.chom.2017.04.011 28494238PMC5490666

[5] : A quantitative imaging-based screen reveals the exocyst as a network hub connecting endocytosis and exocytosis. *Mol Biol Cell*.2015;26(13) : 10.1091/mbc.E14-11-1527 2519-34 10.1091/mbc.E14-11-1527 25947137PMC4571305

[6] : Sec6/8 Complex Is Recruited to Cell–Cell Contacts and Specifies Transport Vesicle Delivery to the Basal-Lateral Membrane in Epithelial Cells. *Cell*.1998;93(5) : 10.1016/S0092-8674(00)81435-X 731-740 10.1016/S0092-8674(00)81435-X 9630218

[7] : The exocyst protein Sec10 is necessary for primary ciliogenesis and cystogenesis in vitro. *Mol Biol Cell*.2009;20(10) : 10.1091/mbc.e08-07-0772 2522-9 10.1091/mbc.e08-07-0772 19297529PMC2682593

[8] : The beta subunit of the Sec61p endoplasmic reticulum translocon interacts with the exocyst complex in Saccharomyces cerevisiae. *J Biol Chem*.2003;278(23) : 10.1074/jbc.M213111200 20946-53 10.1074/jbc.M213111200 12665530

[9] : The exocyst affects protein synthesis by acting on the translocation machinery of the endoplasmic reticulum. *J Biol Chem*.2003;278(23) : 10.1074/jbc.M213210200 20954-60 10.1074/jbc.M213210200 12665531

[10] : Breaching Self-Tolerance to Alu Duplex RNA Underlies MDA5-Mediated Inflammation. *Cell*.2018;172(4) : 10.1016/j.cell.2017.12.016 797-810.e13 10.1016/j.cell.2017.12.016 29395326PMC5807104

[11] : The In Vivo Architecture of the Exocyst Provides Structural Basis for Exocytosis. *Cell*.2017;168(3) : 10.1016/j.cell.2017.01.004 400-412.e18 10.1016/j.cell.2017.01.004 28129539

[12] : Arl13b and the exocyst interact synergistically in ciliogenesis. *Mol Biol Cell*.2016;27(2) : 10.1091/mbc.E15-02-0061 308-20 10.1091/mbc.E15-02-0061 26582389PMC4713133

[13] : The exocyst is a Ral effector complex. *Nat Cell Biol*.2002;4(1) : 10.1038/ncb728 66-72 10.1038/ncb728 11740492

[14] : The exocyst complex binds the small GTPase RalA to mediate filopodia formation. *Nat Cell Biol*.2002;4(1) : 10.1038/ncb720 73-8 10.1038/ncb720 11744922

[15] : The brain exocyst complex interacts with RalA in a GTP-dependent manner: identification of a novel mammalian Sec3 gene and a second Sec15 gene. *J Biol Chem*.2001;276(32) : 10.1074/jbc.C100320200 29792-7 10.1074/jbc.C100320200 11406615

[16] : Coordination of Rab8 and Rab11 in primary ciliogenesis. *Proc Natl Acad Sci U S A*.2010;107(14) : 10.1073/pnas.1002401107 6346-51 10.1073/pnas.1002401107 20308558PMC2851980

[17] : Histone deacetylase is a direct target of valproic acid, a potent anticonvulsant, mood stabilizer, and teratogen. *J Biol Chem*.2001;276(39) : 10.1074/jbc.M101287200 36734-41 10.1074/jbc.M101287200 11473107

[18] : Cdc42 deficiency causes ciliary abnormalities and cystic kidneys. *J Am Soc Nephrol*.2013;24(9) : 10.1681/ASN.2012121236 1435-50 10.1681/ASN.2012121236 23766535PMC3752951

# Peer review: Reviewer response for version 1

**DOI:** 10.21956/wellcomeopenres.16521.r36431

**Published:** 2019-10-08

**Authors:** Viktor Žárský

**Affiliations:** 1 Department of Experimental Plant Biology, Faculty of Science, Charles University, Prague, Czech Republic; 2 Laboratory of Cell Biology, Institute of Experimental Botany CAS, Prague, Czech Republic

Mark Field and colleagues opened the field of phylogenetic analyses of tethering complexes evolution more than ten years ago and this report, using five fold increase of well selected genomes, enhances the resolution of the exocyst status in extant eukaryotes.

 p.4. - In Novick et al. (1980) ^1^ bunch of first sec mutants were described (including sec subunits of the exocyst), but exocyst was stepwise discovered later (culminating in TerBush et al. 1996 ^2^ EMBO J). Similarly on p.7 – interaction of Sec4p with Sec15p containing particle is described in Bowser et al. (1992, JCB)[ref3] not in Novick et al. (1980) ^1^.

 In the description of the complex in the Intro it would be important to mention, that it is effector of both RAB and RHO GTPases, and that also EXO70 contributes a crucial direct interaction with the membrane lipids.

 The extent of the expansion of EXO70 family in land plants is not comparable to any other subunit in any other organism (e.g. 23 in Arabidopsis and 47 in rice) and possibly would be correct to highlight it also in the Abstract (nicely demonstrated by the Fig. 2C).

 Evolution of plant EXO70s was first addressed by Marek Eliáš in Synek et al. (2006) ^4^ clearly indicating deep early land plant EXO70 divergence into three subfamilies. Existence of these three subfamilies was fully corroborated by Cvrčková et al. (2012) ^5^ – liverwort Marchantia has three EXO70 paralogs, each in one subfamily (Cvrčková in Rawat et al. 2017) ^6^. The existence of different plant cell cortical secretory domains and exocyst complexes based on differential EXO70 participation is described in Žárský et al. (2009 ^7^ and 2013 ^8^). Importantly data in plant cells specific transcriptomes and proteomes as well as experimental data clearly indicated that in single plant cell several EXO70 paralogs (and therefore exocyst complexes) might operate – tissue specific expression does not explain full multiplicity of EXO70s in land plants. The major driving force of evolution esp. in EXO70.2 land plants subfamily seems to be competition with parasites.

 Two exocyst subcomplexes (4+4) mostly work together, however work on exocyst in autophagy (Bodemann et al 2011 ^9^; Kulich et al. 2013 ^10^) indicates possibility of functional exocyst subcomplexes specialization.

 “Octamer” instead of octomer – as in Fig. 4 – see root of the tree.

 Typo p. 9 not EXO74 and 80, but EXO70 and 84.

 This bioinformatics analysis highlight post-LECA evolutionary destiny of exocyst vesicle tethering complex marked by both local expansions as well as gene/whole complex losses well correlating with lineage specific biological contexts.

I confirm that I have read this submission and believe that I have an appropriate level of expertise to confirm that it is of an acceptable scientific standard.

## References

[1] : Identification of 23 complementation groups required for post-translational events in the yeast secretory pathway. *Cell*.1980;21(1) : 10.1016/0092-8674(80)90128-2 205-215 10.1016/0092-8674(80)90128-2 6996832

[2] : The Exocyst is a multiprotein complex required for exocytosis in Saccharomyces cerevisiae. *EMBO J*.1996;15(23) :6483-94 8978675PMC452473

[3] : Sec8p and Sec15p are components of a plasma membrane-associated 19.5S particle that may function downstream of Sec4p to control exocytosis. *J Cell Biol*.1992;118(5) : 10.1083/jcb.118.5.1041 1041-56 10.1083/jcb.118.5.1041 1512289PMC2289594

[4] : AtEXO70A1, a member of a family of putative exocyst subunits specifically expanded in land plants, is important for polar growth and plant development. *Plant J*.2006;48(1) : 10.1111/j.1365-313X.2006.02854.x 54-72 10.1111/j.1365-313X.2006.02854.x 16942608PMC2865999

[5] : Evolution of the land plant exocyst complexes. *Front Plant Sci*.2012;3: 10.3389/fpls.2012.00159 159 10.3389/fpls.2012.00159 22826714PMC3399122

[6] : The Physcomitrella patens exocyst subunit EXO70.3d has distinct roles in growth and development, and is essential for completion of the moss life cycle. *New Phytol*.2017;216(2) : 10.1111/nph.14548 438-454 10.1111/nph.14548 28397275

[7] : Exocytosis and cell polarity in plants - exocyst and recycling domains. *New Phytol*.2009;183(2) : 10.1111/j.1469-8137.2009.02880.x 255-72 10.1111/j.1469-8137.2009.02880.x 19496948

[8] : Exocyst complexes multiple functions in plant cells secretory pathways. *Curr Opin Plant Biol*.2013;16(6) : 10.1016/j.pbi.2013.10.013 726-33 10.1016/j.pbi.2013.10.013 24246229

[9] : RalB and the exocyst mediate the cellular starvation response by direct activation of autophagosome assembly. *Cell*.2011;144(2) : 10.1016/j.cell.2010.12.018 253-67 10.1016/j.cell.2010.12.018 21241894PMC3038590

[10] : Arabidopsis exocyst subcomplex containing subunit EXO70B1 is involved in autophagy-related transport to the vacuole. *Traffic*.2013;14(11) : 10.1111/tra.12101 1155-65 10.1111/tra.12101 23944713

# Peer review: Reviewer response for version 1

**DOI:** 10.21956/wellcomeopenres.16521.r36061

**Published:** 2019-09-10

**Authors:** Derek Nolan

**Affiliations:** 1 School of Biochemistry and Immunology, Trinity Biomedical Sciences Institute, Trinity College Dublin, Dublin, Ireland

This study investigates the evolution of the exocyst using comparative genomics, phylogenetics and structure prediction. The focus is the evolutionary flexiblity of the exocyst complex, which was prompted by the initial difficulty in identifying all of the eight canonical exocyst subunits in Trypanosomatids along with the recent discovery of an additional 9 ^th^ component, Exo99, in these organisms. The challenge is to identify those orthologues that have diverged significantly and as well as the implications of this additional 9 ^th^ subunit in African trypanosomes.

 The results, nicely presented and summarised in Figs. 3 & 4, reveal the degree of flexibility in exocyst evolution across eukaryotic lineages. There are examples of expansion, notably Exo70 but also Sec 10, absence/loss of individual components, Sec 8 in Entamoaeba, as well as complete loss in Apicomplexa. The other notable finding is that Exo99 appears to be a taxon-restricted subunit most likely with a distinct structure.

 The message of the study is that the conventional view of the exocyst, as a conserved octameric protein complex, needs to be more nuanced. The authors speculate that subtleties in exocyst complex composition (loss as well as presence of new components) suggest a diversity in exocytosis mechanisms. This seems a reasonable proposition, as pointed out here and elsewhere Apicomplexa possess unique secretory organelles that do not appear to require the canonical tether machinery for biosynthesis, while the presence of Exo99 all kinetoplastids (and also the related bodonids), suggests possibly unique elements in export pathways in these organisms.

I confirm that I have read this submission and believe that I have an appropriate level of expertise to confirm that it is of an acceptable scientific standard.
